# Expression, Purification and Identification of CtCVNH, a Novel Anti-HIV (Human Immunodeficiency Virus) Protein from *Ceratopteris thalictroides*

**DOI:** 10.3390/ijms14047506

**Published:** 2013-04-08

**Authors:** Junbo Sun, Yingjuan Su, Ting Wang

**Affiliations:** 1 State Key Laboratory of Biocontrol, School of Life Sciences, Sun Yat-sen University, Guangzhou 510275, China; E-Mail: dxxsjb@163.com; 2 Institute for Technology Research and Innovation of Sun Yat-sen University, Zhuhai 519000, China; 3 CAS Key Laboratory of Plant Germplasm Enhancement and Specialty Agriculture, Wuhan Botanical Garden, Chinese Academy of Sciences, Wuhan 430074, China

**Keywords:** *Ceratopteris thalictroides*, CtCVNH, anti-HIV protein, prokaryotic expression

## Abstract

CVN (cyanovirin-N) is an anti-HIV protein. CVNH (cyanovirin-N homology) represents its homology. In a previous study, we first reported the full-length sequences of the *CVNH* gene cloned from *Ceratopteris thalictroides*. Based on the finding, the coding sequence of *CtCVNH* was optimized in the study, and then a pET prokaryotic expression vector was constructed. The purification and identification of CtCVNH protein were investigated, as well. SDS-PAGE analysis indicated that a 31 kDa protein was overexpressed and mainly accumulated in the soluble fraction. Only a single protein was obtained after the Ni- nitrilotriacetic acid (NTA) affinity chromatography. The purified protein was identified to be the recombinant CtCVNH by both Western blot and peptide mass fingerprinting analysis.

## 1. Introduction

AIDS is a malignant infectious disease that seriously threatens society and economics in the world. At this time, the chemotherapeutic agents, reverse transcriptase and protease, are used to treat HIV infection by preventing HIV replication in host cells. However, these agents cannot prevent HIV from entering the human body, and the side effects include drug resistance and damage to liver and kidney function. Therefore, it is urgent to develop novel, safe and highly effective anti-HIV agents [1,2].

Cyanovirin-N (CVN), a protein with potent anti-HIV activity, was firstly isolated from *Nostoc elliposporum*[3]. CVN is a 101-amino acid single-chain protein without post-translational modification. The protein contains about two 50-amino acid tandem repeats and two disulfide bond (corresponding to C8–C22 and C58–C73, respectively) [4]. The two CVN monomers form a domain-swapped dimmer [5]. The two carbohydrate binding sites of CVN are symmetrically located within a single protein [6]. Their affinities are very different, and one is significantly higher than the other [6]. CVN can resist detergents, denaturants organic solvents and multiple freeze-thaw cycles [3]. Even boiling at 100 °C cannot damage its anti-HIV activity [3]. CVN successfully blocks the virus-cell fusion process mediated by HIV envelope glycoprotein [3]. *In vitro* studies show that CVN inhibits HIV envelope-mediated cell fusion at nanomolar concentrations by interfering with the interaction between gp120 and cellular receptor CD4 [3,6–8]. Boyd *et al.* (1997) found that CVN possessed high activity against HIV in various target cells, including HIV-1 laboratory strains, RF, IIIB, MN, G910-6, A17, 214, SK1 and 205, G1, and HIV-1 primary isolates WEJQ, VIHU, BAKI, WOME, 89.6, Ba-L, Ada-M and SLKA. In addition, CVN has other antiviral activity, such as Ebola [9] and the influenza virus [10].

CVN homologies (cyanovirin-N homology, CVNH) are composed of the CVNH protein family, which is mainly present in bacteria, fungi and ferns. All CVNH proteins share a common fold structure with CVN [6,11,12]. The anti-HIV domain is extremely conserved among CVNHs, which exhibit similar antiviral activity [11]. CVNH is also indicated to be a potential natural anti-HIV protein.

In the previous study, we first reported the full-length genomic DNA of the CVNH gene from *Ceratopteris thalictroides*[12]. In the present, the gene encoding CVNH from *C. thalictroides* (CtCVNH) was optimized and constructed the expression plasmid pET32a-CtCVNH. The plasmid was utilized for expression, purification and identification, which composed a solid foundation for further activity investigation of CtCVNH.

## 2. Results

### 2.1. Optimization of the *CtCVNH* Gene

**Figure 1:**
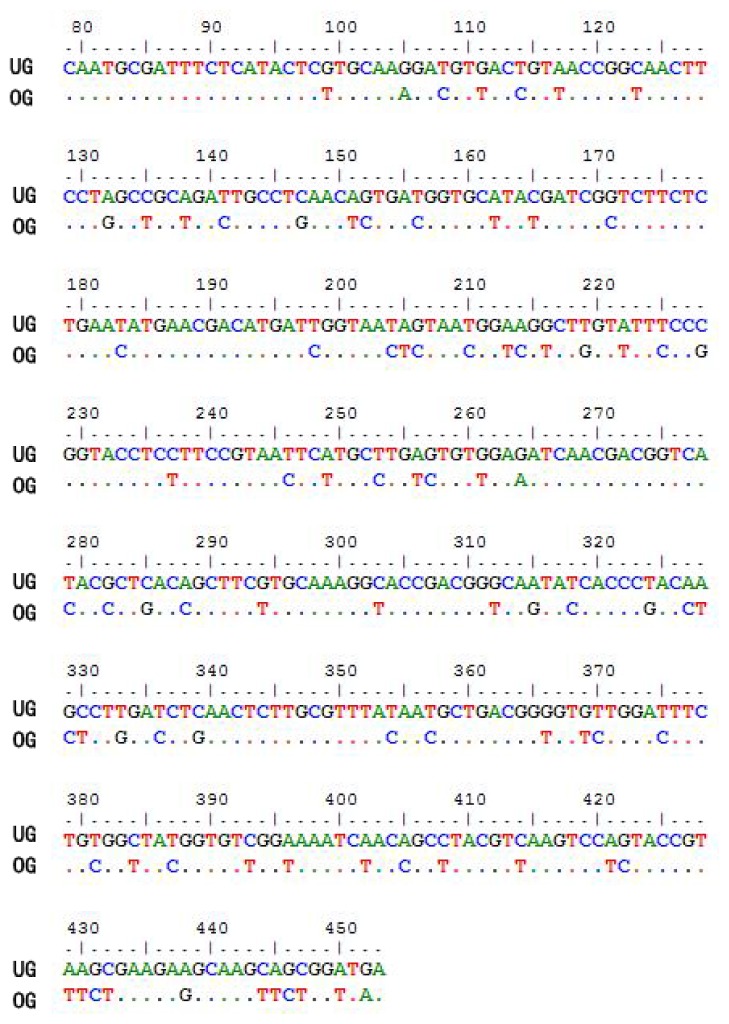
Result of sequence optimization (numbering according to the wild-type coding sequence of cyanovirin-N homology from *C. thalictroides* (CtCVNH)). UG: the wild-type coding sequence of CtCVNH. OG: the optimized coding sequence of CtCVNH.

The *CtCVNH* gene was successfully optimized by deleting a signal peptide-like sequence and the stable secondary structures and regions that may block the ribosome binding site (Figure 1). The constructed plasmid, pET32a-CtCVNH, verified by DNA sequencing, was transformed into *E. coli* JM109.

### 2.2. Construction of the Expression Strain

**Figure 2:**
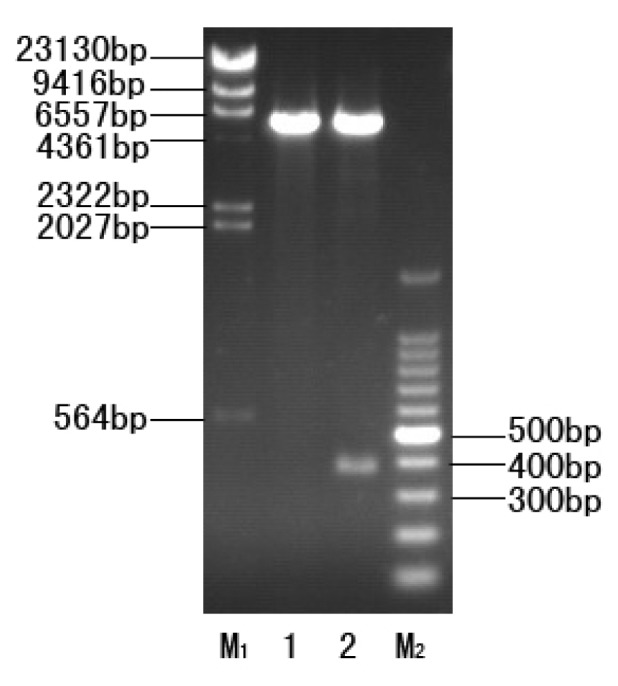
Restriction enzyme digestion of the recombinant plasmid pET32a-CtCVNH by EcoR I and Hind III. M_1_: λ-*H*ind III digest DNA marker. 1, pET32a (+); 2, pET32a-CtCVNH; M_2_, 100 bp DNA ladder marker.

The optimized *CtCVNH* gene was confirmed to be successfully inserted into the expression vector pET32 (+). The recombinant plasmid pET32a-CtCVNH digested with EcoR I and Hind III corresponding to 5900 bp and 375 bp bands, respectively (Figure 2). The inserted DNA sequence was identical to the optimized *CtCVNH* gene after having been successfully transformed into Rosetta 2 (DE3).

### 2.3. Protein Expression and Solubility Analysis

**Figure 3:**
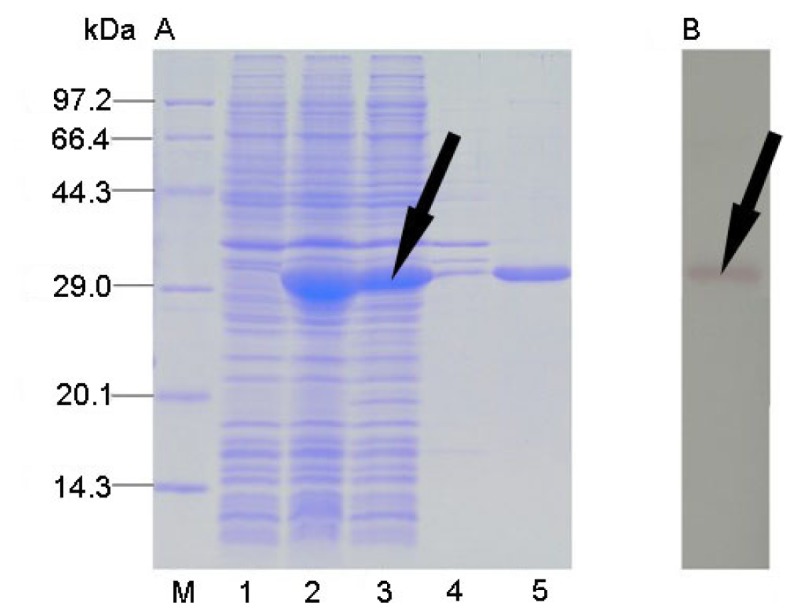
Coomassie-stained SDS–PAGE gel (**A**) and Western blot (**B**) showing the expression profile of CtCVNH. M, low molecular protein marker; Lane 1, sample before induction; Lane 2, sample after induced; Lane 3, supernatant; Lane 4, inclusion body; Lane 5, purified sample.

Recombinant *CtCVNH* gene was expressed in Rosetta 2 (DE3) induced by 0.5 mM IPTG for 2 h. The recombinant protein corresponded to 31 kDa, suggesting an efficient expression of the optimized *CtCVNH* gene. Solubility analysis indicated that CtCVNH protein accumulated mainly in the soluble fraction (Figure 3A).

### 2.4. Protein Purification and Western Blot Analysis

A single protein band with correct molecular weights from the supernatant was obtained by Ni-NTA resin purification (Figure 3A). The results also indicated that the recombinant CtCVNH is of high purity. The purified proteins were further confirmed by Western blot with mouse anti-His tag monoclonal antibody and goat anti-mouse IgG antibody conjugated to alkaline phosphatase, which detected a single band with correct molecular weights (Figure 3B).

### 2.5. PMF Analysis and Molecular Weight Determination

**Figure 4:**
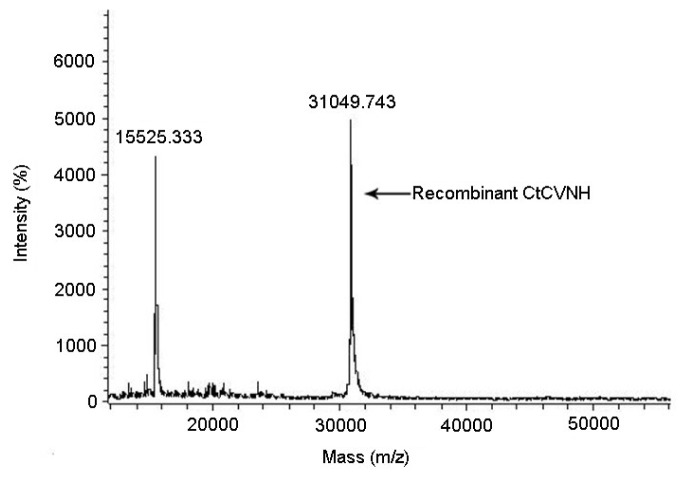
The molecular weight of recombinant CtCVNH determined by MATOL-TOF/MS.

The recombinant protein matched well with the *C. richardii* CVNH data set (GenBank Accession No. BQ087187, Score 282, *p* < 0.05). The *m*/*z* ratios yielded experimental molecular weights of 31,049.74 Da for recombinant protein, which was close to the predicted mass of 31,048.83 Da (Figure 4).

## 3. Discussion

*Escherichia coli* is one of the most favorite hosts for protein heteroexpression. It is widely used in protein expression, due to its low cost, fast growth and high yield [13]. Although many proteins have been successfully expressed in *E. coli*, most of them were expressed as inactive inclusion bodies [13]. The purification of inclusion bodies is considered labor-intensive, time-consuming and not cost-effective [13]. The pET systems are very powerful for high efficient prokaryotic expression under the control of strong bacteriophage T7 transcription and translation signals. Vector pET32a (+) is one of the pET systems that permits target genes to be fused to trxA for high levels of expression with good solubility [14].

In a previous study, we constructed a recombinant pET32a (+) plasmid to produce wild-type CtCVNH. The recombinant protein CtCVNH could be expressed in *E. coli*. Several *E. coli* rare codons were found in the coding sequence of CtCVNH, two of which were in tandem. In order to avoid translation errors caused by rare codons, *E. coli* strain Rosetta 2 (DE3), which could supply tRNAs recognizing rare codons, was used as the expression host for recombinant CtCVNH. However, two expression proteins were detected, corresponding to 34 kDa and 23 kDa, respectively, which could not be separated by affinity chromatography. The Peptide Mass Fingerprinting (PMF) and bioinformatics analysis indicated that the 23 kDa protein was identical to the N-terminus part of the target protein. We inferred that the 23 kDa protein was a partial product resulting from premature termination [15,16].

In order to obtain the single recombinant CtCVNH protein, the coding sequence of the CtCVNH protein was optimized. Codons that might affect protein expression were replaced with *E. coli* preferred codons. The protein of the optimized gene was expressed as described above. An obvious expression band about 31 kDa could be detected. More importantly, only a target protein was detected, which was purified through affinity chromatography. The purified protein was further confirmed by Western blot and PMF to be the recombinant CtCVNH. Due to Trx tag attached to the *N*-terminus of the protein, the target protein can be directly purified from the supernatant. Therefore, the denaturation and refolding of the protein can be avoided. Furthermore, as a native protein of *E. coli*, Trx can protect CtCVNH from protease degradation [17].

## 4. Experimental Section

### 4.1. Experimental Materials

Plasmid pET32a (+) and *E. coli* strain Rosetta 2 (DE3) were used as hosts for the expression of CVNH. λ-Hind III digest DNA marker, 100 bp DNA ladder marker, protein molecular weight marker (low) and restriction enzymes EcoR I and Hind III were supplied by TaKaRa (Dalian, China). An AxyPrepTM Plasmid Miniprep Kit was purchased from Axygen (Hangzhou, China). Ni-NTA His·Bind Resin was obtained from Novagen (San Diego, CA, USA). Amicon Ultra-15 centrifugal filter units (10 kDa) were acquired from Millipore (Billerica, MA, USA). Mouse anti-His6 monoclonal antibody and goat anti-mouse IgG antibody conjugated to alkaline phosphatase (AP) were purchased from CWBIC (Beijing, China). All other chemicals were of analytical grade and obtained from commercial sources.

### 4.2. Sequence Optimization and Construct Design

Based on the previous study, the gene encoding CtCVNH was obtained from its full length sequences [12]. First, the signal peptide was predicted using SignalP 4.0 [18] (http://www.cbs.dtu.dk/services/SignalP/). Second, after the signal peptide was deleted, the *CtCVNH* gene was optimized by Jcat, which is a web-based program [19] (http://www.jcat.de/). Third, the optimized *CtCVNH* gene was inserted into plasmid pET32a (+) between the digestive sites, EcoR I and Hind III. Fourth, the secondary structure of the mRNA of the expression plasmid was predicted by RNAfold (http://rna.tbi.univie.ac.at/) [20]. Fifth, the *CtCVNH* gene was further optimized to eliminate the stable secondary structures and regions that might block the ribosome binding site. Finally, the finial optimized *CtCVNH* gene introduced to the sites of EcoR I and Hind III was synthesized in TaKaRa, which was ligated to the similarly digested expression vector, pET32a (+). The constructed recombinant plasmid pET32a-CtCVNH was transformed into *E. coli* JM109.

### 4.3. Construction of the Expression Strain

Plasmid DNA from the successful clones of pET32a-CtCVNH was extracted using the AxyPrep™ Plasmid Miniprep Kit and verified by restriction enzyme digestion. The plasmid DNA was transformed into Rosetta 2 (DE3) *E. coli* cells for expression of the recombinant protein. The sequence of the cloned gene was verified by sequencing. The plasmid was stored at −80 °C for glycerol stocks.

### 4.4. Expression of Recombinant Protein and Solubility Analysis

A single positive pET32a-CtCVNH clone was inoculated into 10 mL LB culture containing ampicillin (100 μg/mL) and chloramphenicol (34 μg/mL) and incubated overnight at 37 °C. The overnight culture was inoculated into 300 mL fresh LB culture containing ampicillin and chloramphenicol at a ratio of 1:100 and grown at 37 °C with shaking (220 rpm). When the OD_600_ reached approximately 0.6–1.0, isopropyl β-D-1-thiogalactopyranoside (IPTG) was added with a final concentration of 0.2 mM. The culture was incubated at 37 °C for an additional 2 h, and the protein was autoinduced to be expressed during this period.

### 4.5. Protein Purification

The cells were harvested from the 300 mL culture by centrifugation at 8,000× *g* for 6 min at 4 °C. Then, the cells were resuspended in 15 mL of lysis buffer (20 mM Tris-HCL, pH 7.5), and then lysed on ice by sonication at 30 W for 50 cycles (10 s working, 15 s free). The pellet and the supernatant were separated by centrifugation at 14,000× *g* for 20 min. The pellet was resuspended in 30 mL buffer with 20 mM Tris-HCl (pH 7.5) to dissolve the expression protein. Each fraction was analyzed using 15% standard SDS-PAGE gel electrophoresis. The recombinant proteins were purified using Novagen Ni-NTA chromatography (San Diego, CA, USA), according to the manufacturer’s protocols. Firstly, Ni-NTA His·Bind Resin was washed with 5-times the volume of 20 mM Tris (pH 7.5) and balanced overnight at 4 °C. The sample was loaded onto the column after passing through a 0.45 μm syringe filter. Secondly, unbound proteins were removed with 10-times the volume of Buffer I (10 mM Tris-HCl, 250 mM NaCl, 2.5 mM imidazole, pH 7.5), whereas the recombinant proteins were eluted with 5-times the volume of Buffer I (containing 50, 100 and 500 mM imidazole). Finally, the column was eluted with 5-times the volume of Buffer II (20 mM Tris-HCl, 500 mM NaCl, 100 mM EDTA, pH 7.5).

### 4.6. Western Blot Analysis

Electrophoretic separation of protein preparations was carried out using SDS-PAGE. The proteins were transferred to nitrocellulose membranes by electroblotting. The membrane was blocked with 3% BSA at room temperature for 2 h and was incubated overnight with mouse anti-His tag monoclonal antibody (1:5,000). After washing, the membrane was incubated with goat anti-mouse IgG antibody conjugated to alkaline phosphatase (1:10,000) for 2 h at room temperature.

### 4.7. Peptide Mass Fingerprinting Analysis and Molecular Weight Determination

The Peptide Mass Fingerprinting (PMF) and Matrix Assisted Laser Desorption/Ionization Time of Flight Mass Spectrometry (MALDI-TOF/MS) were performed on MLtraflex MALDI-TOF/TOF (Bruker Corporation, Billerica, MA, USA) in the Experimental Center, School of Life Science, Sun Yat-sen University (Guangzhou, China). For PMF, the purified recombinant protein was electrophoresed on SDS-PAGE, followed by Coomassie staining. The protein band was carefully excised, destained and digested using trypsin. Data were analyzed using software Mascot v2.3 by searching for CVNH in the NCBI non-redundant database. For MALDI-TOF/MS, 5 mL of purified protein was added to the Amicon Ultra-15 centrifugal filter units (10 kDa) and spun at 3,500× *g* for 40 min at 4 °C. Ten milliliters of ultra-pure water were added and spun at 3,500× *g* for 60 min at 4 °C and repeated once. The samples were then analyzed under high vacuum with a 25 kV beam. All MS spectra were acquired in positive-ion mode with 200 laser pulses per sample spot.

## 5. Conclusions

This study is the first to report the construct of a pET prokaryotic expression vector based on the optimized coding sequence of *CtCVNH*. CVNH protein can be successfully expressed, purified and identified, which lays a solid foundation for further activity investigation of CtCVNH. In another experiment, we optimized the culture condition of CVNH protein expression, including the type and the ingredients of the culture medium, initial pH, OD value, induction agents and concentrations, induction time and expression time, respectively [21]. In the future, we will explore the biologically active, clinical trials, large-scale preparation and commercialization of CtCVNH.
